# Multicenter retrospective comparison of safety and efficacy among three antithrombotic regimens following TAVI

**DOI:** 10.3389/fphar.2025.1531361

**Published:** 2025-06-11

**Authors:** Yu Ren, Jing Chen, Suchun Wang, Zhengli Jiang, Hua Luo

**Affiliations:** ^1^ Department of Pharmacy, Taizhou Hospital of Zhejiang Province affiliated to Wenzhou Medical University, Taizhou, Zhejiang, China; ^2^ Department of Orthopedic, Taizhou Hospital of Zhejiang Province affiliated to Wenzhou Medical University, Linhai, China

**Keywords:** TAVI, antithrombotic therapy, dual antiplatelet therapy, warfarin, postoperative complications

## Abstract

**Objective:**

This study comprehensively evaluates the safety and efficacy of three antithrombotic regimens following Transcatheter Aortic Valve Implantation (TAVI), focusing on thrombotic and bleeding complications to provide data-driven insights for optimizing postoperative management.

**Methods:**

A retrospective cohort analysis included 58 TAVI patients from two medical centers (from August 2022 to July 2024). Patients were assigned to three regimens post-TAVI: Group A (warfarin for 3–6 months transitioned to lifelong aspirin), Group B (warfarin transitioned to rivaroxaban), and Group C (dual antiplatelet therapy transitioned to aspirin). Key exclusion criteria were concurrent cardiac surgeries and severe hepatic or renal dysfunction. Primary outcomes included transfusion rates, bleeding incidents, and thrombotic events. Secondary outcomes included coagulation parameters [international normalized ratio (INR), prothrombin time (PT), activated partial thromboplastin time (APTT), D-dimer] and postoperative hospital stay duration.

**Results:**

Transfusion requirements did not differ significantly across groups (p = 0.576). However, significant differences were noted in bleeding events (p = 0.034) and hospital stay duration (p < 0.001) among groups. Group B (warfarin transitioned to rivaroxaban) had the lowest bleeding incidence (0%) and the shortest hospital stay (8.71 ± 3.58 days), compared to Group A (37.5%, 14.71 ± 7.61 days) and Group C (30.0%, 7.50 ± 2.84 days). Transfusion requirements and thrombotic event rates were comparable across groups. APTT was significantly prolonged in Group C (p < 0.001), without corresponding clinical bleeding.

**Conclusion:**

Each antithrombotic regimen presented unique clinical benefits and limitations. Transitioning from warfarin to rivaroxaban was associated with a significantly reduced risk of bleeding and shorter hospital stays. Transitioning from dual antiplatelet therapy to aspirin monotherapy significant prolonged APTT without increasing clinical bleeding events. These findings highlight the importance of tailored antithrombotic strategies to optimize post-TAVI outcomes.

## Introduction

Aortic stenosis, a progressive valvulopathy prevalent in aging populations, imposes substantial hemodynamic burden that culminates in heart failure, syncope, or sudden cardiac death without intervention (Taniguchi et al., 2018; Otto et al., 2020b). Surgical aortic valve replacement (SAVR) has long been the standard of care; however, its high perioperative risks often preclude its use in elderly patients with multiple comorbidities (Chen et al., 2024; Ma et al., 2024). The advent of transcatheter aortic valve implantation (TAVI) in 2002 revolutionized treatment paradigms through a minimally invasive approach, demonstrating superior procedural safety and accelerated recovery (Cribier et al., 2002; Blankenberg et al., 2024). Nevertheless, post-TAVI antithrombotic management remains clinically contentious, particularly in elderly cohorts where concomitant atrial fibrillation and coronary artery disease amplify thromboembolic-hemorrhagic risk duality (Atkins and Reardon, 2024; Ajmal et al., 2024).

Current clinical guidelines provide limited consensus on the optimal post-TAVI anticoagulation regimen, reflecting the complexity of this issue (Otto et al., 2020a; Baumgartner et al., 2017; Vahanian et al., 2021). Commonly utilized strategies include vitamin K antagonists (VKAs), such as warfarin non-vitamin K antagonist oral anticoagulants (NOACs), and antiplatelet therapies. Each therapeutic pathway presents distinct trade-offs, with varying effects on thrombotic and bleeding risks, further complicating clinical decision making.

The lack of robust, head-to-head comparative studies evaluating the safety and efficacy of these regimens in the TAVI population further exacerbates these challenges. Real-world data on their relative impact on major adverse events, including bleeding, thromboembolism, and mortality, remain limited (Didier et al., 2021; Kosmidou et al., 2019). Our multicenter retrospective analysis addresses this evidence gap through comparative evaluation of three sequential therapeutic sequences: warfarin followed by aspirin, warfarin followed by rivaroxaban, and dual antiplatelet therapy transitioning to aspirin. The investigation seeks to elucidate regimen specific safety profiles and thromboprophylactic efficacy to facilitate personalized postoperative management in contemporary TAVI practice.

## Methods

### Study design

This retrospective cohort study was conducted at two tertiary medical centers and included patients who underwent TAVI from 1 August 2022 through 31 July 2024. Patients were stratified into three groups based on the antithrombotic regimen prescribed post-TAVI: Group A (warfarin for 3–6 months followed by lifelong aspirin), Group B (warfarin for 3–6 months followed by rivaroxaban), and Group C (dual antiplatelet therapy with aspirin and clopidogrel/ticagrelor transitioning to single antiplatelet therapy with aspirin).

Therapeutic selection was determined by the treating physician based on clinical evaluation incorporating cardiac rhythm status, including atrial fibrillation, preprocedural anticoagulation/antiplatelet exposure, renal function, and individualized bleeding risk assessment. To reduce potential selection bias and ensure baseline comparability among groups, we systematically evaluated demographic and clinical characteristics, including age, sex, major comorbidities (diabetes, prior stroke), and pre-existing thrombotic prevention regimens. For atrial fibrillation subgroups, stroke and bleeding risk were assessed using the CHA_2_DS_2_-VA and HAS-BLED risk stratification instruments, respectively. Notably, the CHA_2_DS_2_-VA score, as recommended by the 2024 European Society of Cardiology (ESC) guidelines for atrial fibrillation management, represents a simplified version of the conventional CHA_2_DS_2_-VASc model, with sex removed as a contributing factor (Van Gelder et al., 2024). A comprehensive summary of baseline variables and risk stratification scores is presented in Table 1.

**TABLE 1:** Baseline characteristics.

Group	Group A (n = 24) (Warfarin → aspirin)	Group B (n = 14) (Warfarin → rivaroxaban)	Group C (n = 20) (DAPT → aspirin)	p-value
Age (median, IQR)	71 (66, 73.3)	74 (70.8, 76.3)	74.5 (72, 78)	0.078
Sex (Male/Female)	14/10	8/6	10/10	0.846
Atrial Fibrillation (n, %)	4 (16.67)	4 (28.57)	4 (20)	0.378
Stroke (n, %)	7 (29.16)	3 (21.43)	6 (30)	0.145
History of Bleeding (n, %)	1 (4.17)	0 (0)	1 (5)	0.373
Prior Antithrombotic Use (n, %)	5 (20.83)	5 (35.71)	0 (0)	0.138
Aspirin (n, %)	1 (4.17)	2 (14.28)	0 (0)	—
Clopidogrel (n, %)	1 (4.17)	0 (0)	0 (0)	—
DAPT (n, %)	1 (4.17)	2 (14.28)	0 (0)	—
Warfarin (n, %)	0 (0)	1 (7.14)	0 (0)	—
NOAC (n, %)	2 (8.33)	0 (0)	0 (0)	—
CHA_2_DS_2_-VA^a^ (median, IQR)	2.0 (1.75–2.25)	3.0 (2.75–3.25)	2.5 (1.75–3.25)	0.360
HAS-BLED^a^ (median, IQR)	1.5 (0.75–2.0)	0.5 (0.0–1.25)	1.0 (0.75–1.5)	0.718

^a^ CHA_2_DS_2_-VA, and HAS-BLED, scores were calculated only for patients with atrial fibrillation (AF) in the study cohort.

It is noteworthy that all TAVI procedures in this cohort utilized self-expanding valves, as balloon-expandable valves were not employed at either institution during the study period. Eligible patients were required to have completed the TAVI procedure and possess comprehensive follow-up records, including coagulation parameters and clinical outcomes. Patients were excluded if they underwent concurrent cardiac surgeries or had severe hepatic or renal dysfunction. Ethics approval was obtained from the institutional review board at both centers (Approval No.: L20240902).

### Outcomes

Primary outcomes were the incidence of transfusions, hemorrhagic events, and thrombotic complications. Hemorrhagic events were classified using the Bleeding Academic Research Consortium (BARC) criteria: minor bleeding (BARC 0–2), major bleeding (BARC 3–4), and fatal bleeding (BARC 5).

Thrombotic events included ischemic stroke and venous thromboembolism (VTE). Ischemic stroke was defined as new-onset focal neurological deficits confirmed by head computed tomography (CT) or magnetic resonance imaging (MRI). VTE comprised deep vein thrombosis and pulmonary embolism, diagnosed using duplex ultrasonography or computed tomography pulmonary angiography. Notably, no systematic screening for subclinical thrombotic events (e.g., silent stroke or valve thrombosis) was performed; therefore, only clinically manifest events were recorded. Secondary outcomes included coagulation parameters [international normalized ratio (INR), prothrombin time (PT), activated partial thromboplastin time (APTT), and D-dimer levels] and the duration of hospitalization.

### Statistical analysis

All statistical analyses were performed using SPSS software (version 26.0; IBM Corp., Armonk, NY, United States), and R software (version 4.3.1; R Foundation for Statistical Computing, Vienna, Austria). Descriptive statistics were used to summarize baseline demographic and clinical characteristics.

Continuous variables were presented as mean ± standard deviation or median (interquartile range) based on data distribution and compared using the Kruskal–Wallis H test due to non-parametric distribution. Categorical variables were expressed as frequencies and percentages and analyzed using the Chi-square test. CHA_2_DS_2_-VA and HAS-BLED scores, were also analyzed using the Kruskal–Wallis H test. Kaplan-Meier survival analysis was utilized to assess the cumulative incidence of bleeding and thrombotic events across groups, and differences were evaluated with the log-rank test. To address the potential bias arising from small sample size and rare event rates, Firth’s penalized logistic regression was employed to estimate adjusted odds ratios (ORs) and 95% confidence intervals (CIs) for bleeding and thrombotic outcomes, comparing Group B and Group C with Group A as the reference. Adjustment variables included age, sex, and atrial fibrillation status.

Non-inferiority margins were prespecified based on prior landmark cardiovascular and anticoagulation studies, including PLATO, GALILEO, and RE-ALIGN trials (Wallentin et al., 2009; Dangas et al., 2020; Eikelboom et al., 2013). Non-inferiority was defined as an upper 95% CI < 1.25 for bleeding events and <1.38 for thrombotic events. All p-values were two-sided, and statistical significance was defined as p < 0.05.

## Results

**FIGURE 1:**
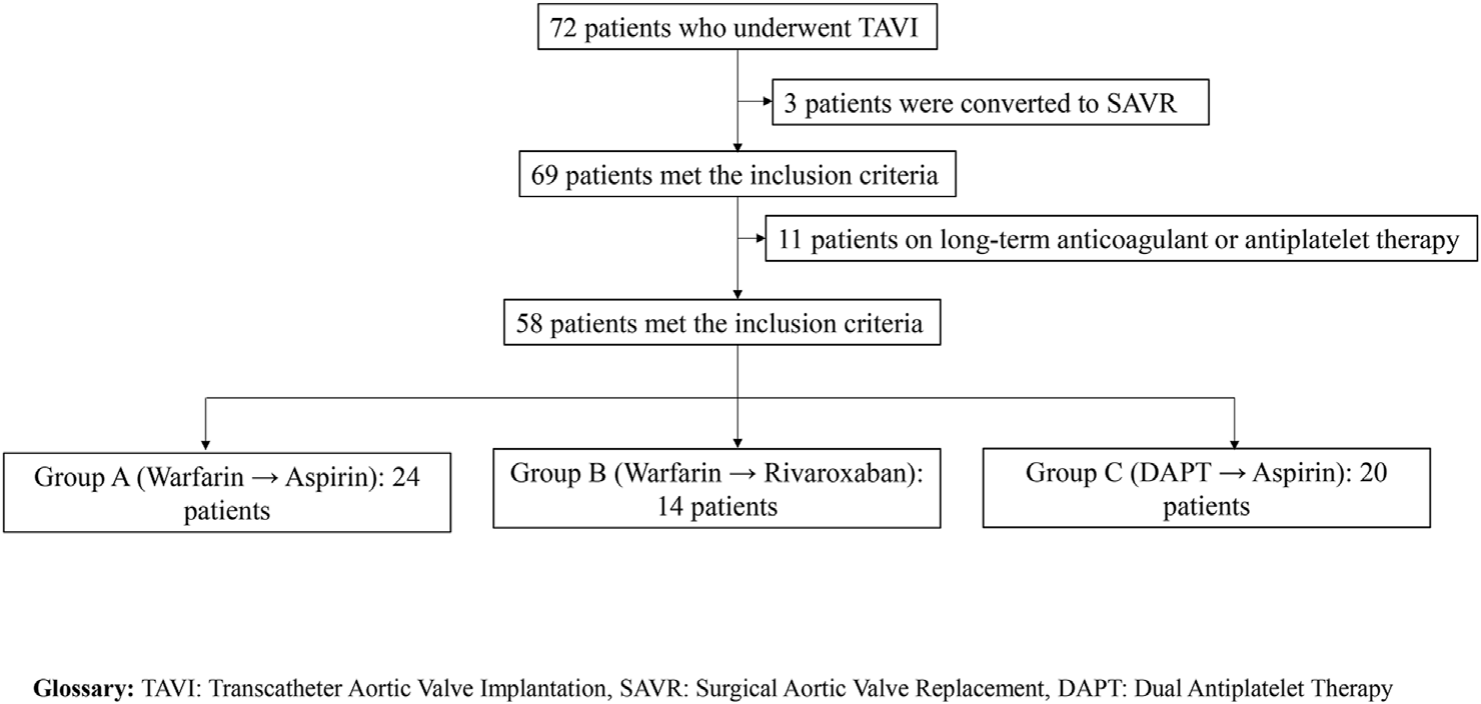
Patient selection process.

A total of 58 patients were included in this study and stratified into three groups based on post-TAVI anticoagulation regimens: Group A (warfarin for 3–6 months followed by lifelong aspirin, n = 24), Group B (warfarin transitioned to rivaroxaban, n = 14), and Group C (dual antiplatelet therapy with aspirin and clopidogrel/ticagrelor transitioning to single-agent aspirin, n = 20). A detailed flowchart of the patient selection process is provided in Figure 1. Clinical outcomes, hematologic parameters, coagulation indicators, and postoperative hospital stay were evaluated and summarized in Table 1.

### Primary outcomes

#### Bleeding and transfusion events

The incidence of bleeding events differed significantly among the three groups. Bleeding occurred in 9 of 24 patients in Group A (37.5%, 95% CI: 18.8%–59.4%), 0 of 14 in Group B (0%, 95% CI: 0%–23.2%), and 6 of 20 in Group C (30.0%, 95% CI: 11.9%–54.3%) (Chi-square p = 0.034). Major bleeding events were reported in 2 patients each in Group A (8.3%) and Group C (10.0%), while Group B reported no major bleeding. One fatal intracranial hemorrhage occurred in Group C (5.0%) (detailed data are presented in Table 2).

**TABLE 2:** Clinical outcomes.

Outcomes	Group A (n = 24) (Warfarin → aspirin)	Group B (n = 14) (Warfarin → rivaroxaban)	Group C (n = 20) (DAPT → aspirin)	p-value
Primary outcomes				
Tansfusions, %	11 (45.83)	4 (28.57)	8 (40)	0.576
Hmorrhagic events, %	9 (37.5)	0 (0)	6 (30)	0.034
Minor bleeding, %	7 (29.17)	0 (0)	3 (15)	0.086
Major bleeding, %	2 (8.33)	0 (0)	2 (10)	0.361
Fatal bleeding, %	0 (0)	0 (0)	1 (5)	0.251
Thrombotic events, %	5 (20.83)	2 (14.29)	2 (10)	0.607
Ischemic stroke, %	1 (4.17)	2 (14.29)	0 (0)	0.173
VTE, %	4 (16.67)	0 (0)	2 (10)	0.266
Secondary Outcomes				
RBC, 10^12^/L	3.51 ± 0.54	3.44 ± 0.58	3.16 ± 0.55	0.115
Hb, g/L	107.33 ± 16.15	100.29 ± 10.28	97.3 ± 15.68	0.079
PLT	130.42 ± 60.50	122.00 ± 45.39	126.50 ± 37.52	0.881
INR	1.22 ± 0.20	1.18 ± 0.05	1.18 ± 0.13	0.572
PT, s	15.22 ± 1.86	14.83 ± 0.49	14.77 ± 1.16	0.52
APTT, s	35.74 ± 3.45	38.16 ± 3.50	41.49 ± 4.64	<0.001
D-dimer (mg/L)	1.15 ± 0.97	1.99 ± 2.16	1.44 ± 0.67	0.159
Postoperative Hospital Stay, days	14.71 ± 7.61	8.71 ± 3.58	7.50 ± 2.84	<0.001

RBC: red blood cell count, Hb: Hemoglobin, PLT: platelet count, INR: international normalized ratio, PT: prothrombin time, APTT: activated partial thromboplastin time, DAPT: dual antiplatelet therapy, BARC: bleeding academic research consortium, VTE: venous thromboembolism.

**FIGURE 2:**
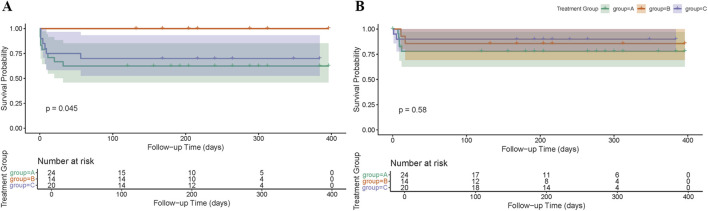
Cumulative incidence of bleeding and thrombotic events among post-TAVI patients stratified by antithrombotic regimen. **(A)** Kaplan–Meier curves depicting the cumulative incidence of bleeding events. **(B)** Kaplan–Meier curves depicting the cumulative incidence of thrombotic events.

Kaplan-Meier survival analysis confirmed that Group B had a significantly lower cumulative incidence of bleeding events compared to Groups A and C over time (log-rank p = 0.045; Figure 2A). All bleeding events were confined to Groups A and C.

**FIGURE 3:**
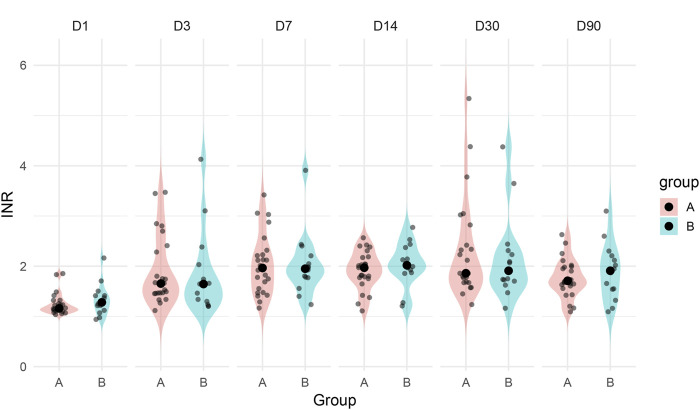
INR variation from postoperative day 1 to day 90 by treatment group.

Among patients in Group A, both major bleeding episodes occurred in individuals with supratherapeutic INR levels (>3.5), suggesting that excessive anticoagulation may have contributed to hemorrhagic risk. In contrast, patients in Group B maintained stable INR values during the warfarin treatment phase and transitioned to rivaroxaban without bleeding complications. This observation is further supported by longitudinal INR data across postoperative days 1–90, which demonstrated greater variability and higher outliers in Group A (Figure 3). The visual pattern of INR instability in Group A aligns with its higher bleeding incidence, reinforcing the clinical relevance of optimal anticoagulation control in the early postoperative period.

Transfusion requirements were comparable across the groups and did not reach statistical significance (p = 0.576). Transfusions were administered in 11 of 24 patients in Group A (45.8%, 95% CI: 25.6%–67.2%), 4 of 14 in Group B (28.6%, 95% CI: 8.4%–58.1%), and 8 of 20 in Group C (40.0%, 95% CI: 19.1%–63.9%). Although the difference was not statistically significant, the higher transfusion rate in Group A was consistent with its increased bleeding incidence, further supporting the association between supratherapeutic INR and bleeding burden.

Most bleeding events occurred within the first 60 postoperative days, with no new events observed beyond day 90. The time-to-event curves suggest that Group B maintained a consistently lower cumulative incidence throughout the follow-up period (Figure 2A).

#### Thrombotic events

Thrombotic events were relatively infrequent and evenly distributed among the groups: 5 of 24 patients in Group A (20.8%, 95% CI: 7.1%–42.2%), 2 of 14 in Group B (14.3%, 95% CI: 1.8%–42.8%), and 2 of 20 in Group C (10.0%, 95% CI: 1.2%–31.7%). These included ischemic strokes in 1 (Group A), 2 (Group B), and 0 (Group C) patients, and VTEs in 4 (Group A), 0 (Group B), and 2 (Group C) patients.

Kaplan-Meier analysis showed no significant difference in cumulative incidence or event timing among the three groups (log-rank p = 0.58; Figure 2B). Similarly, thrombotic events in all groups occurred early, with no new events detected after postoperative day 100. These findings emphasize that the critical window for both bleeding and thrombotic complications is concentrated within the early postoperative phase.

### Secondary outcomes hematologic and coagulation parameters

There were no significant differences in red blood cell count (RBC, p = 0.115), hemoglobin (Hb, p = 0.079), or platelet count (PLT, p = 0.881) across the three groups. Similarly, PT and INR were comparable among groups (p = 0.520 and p = 0.572, respectively).

However, a significant difference was noted in APTT (p < 0.001), with Group C showing the highest values (41.49 ± 4.64 s), followed by Group B (38.16 ± 3.50 s) and Group A (35.74 ± 3.45 s). In Group C (DAPT transitioning to aspirin), APTT on postoperative day 3 was significantly prolonged compared to the other groups (p = 0.024), see Table 2 for details. This finding may reflect a transient alteration in coagulation dynamics due to postoperative inflammatory responses or perioperative antiplatelet exposure. However, the prolongation was not accompanied by an increased incidence or severity of clinical bleeding, suggesting that the observed APTT elevation did not translate into a higher bleeding risk in this context. As APTT is not routinely used to monitor antiplatelet therapy, its prognostic value in this setting remains unclear. Further mechanistic or pharmacodynamic studies are warranted to determine whether isolated postoperative APTT changes have any clinical significance in patients undergoing TAVI.

#### Length of hospital stay

**FIGURE 4:**
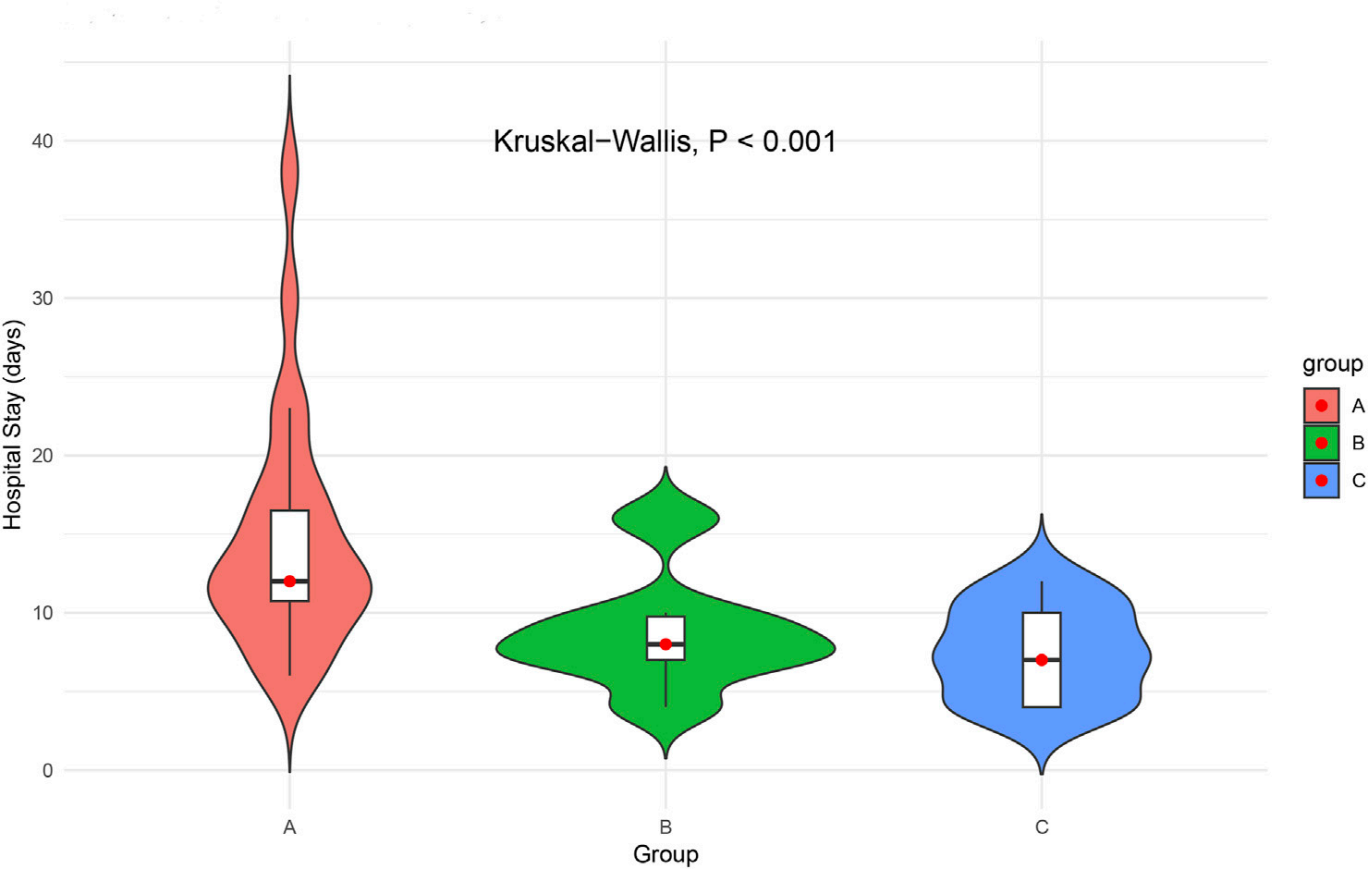
Postoperative hospital stay duration by antithrombotic regimen.

The duration of postoperative hospitalization varied significantly across the three groups (Kruskal–Wallis p < 0.001). Patients in Group A had the longest average hospital stay (14.71 ± 7.61 days; Figure 4). Group A patients had the longest hospital stay, with a median of 12.0 days (IQR: 10.75–16.50), compared to 8.0 days (IQR: 7.00–9.75) in Group B and 7.0 days (IQR: 4.00–10.00) in Group C. The prolonged hospitalization observed in Group A likely reflects the clinical burden of bleeding events and associated management. These findings underscore the importance of balancing antithrombotic efficacy with bleeding risk in optimizing postoperative recovery and resource utilization.

#### Noninferiority comparison of bleeding and thrombotic events

To address potential bias caused by rare events and separation, Firth’s penalized logistic regression was employed to compare the adjusted odds of bleeding and thrombotic complications across groups.

For bleeding events, compared with Group A (warfarin followed by aspirin), Group B (warfarin transitioned to rivaroxaban) showed a significantly lower bleeding risk (OR = 0.063, 95% CI: 0.001–0.861, p = 0.044), suggesting both non-inferiority and superiority. Group C (DAPT transitioned to aspirin) was also non-inferior to Group A (OR = 0.220, 95% CI: 0.042–1.075), although the difference was not statistically significant (p = 0.062).

**FIGURE 5:**
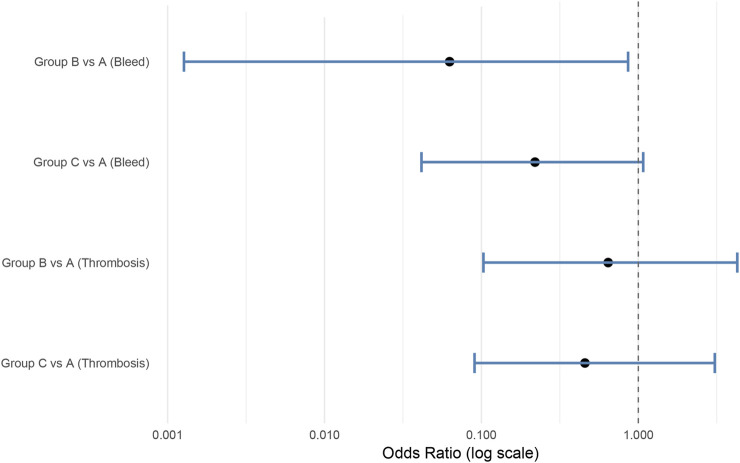
Forest plot illustrating adjusted odds ratios (OR) and 95% confidence intervals (CI) for bleeding and thrombotic events following TAVI. Analysis based on Firth logistic regression. Group A served as the reference.

For thrombotic events, Group B had lower odds of thrombosis (OR = 0.643, 95% CI: 0.103–4.277, p = 0.642), and Group C also showed a numerical reduction (OR = 0.457, 95% CI: 0.090–3.076, p = 0.417). However, the wide confidence intervals and upper limits exceeding the prespecified non-inferiority margin of 1.38 indicate that non-inferiority could not be confirmed for thrombotic outcomes. A summary forest plot of the adjusted odds ratios and 95% confidence intervals is presented in Figure 5.

#### Summary of findings

Significant differences in bleeding incidence, coagulation parameters, and postoperative hospital stay were observed among the three post-TAVI antithrombotic strategies. Patients in Group B (warfarin transitioned to rivaroxaban) exhibited the lowest bleeding rates, shortest hospital stay, and greater INR stability during the anticoagulation phase, with no supratherapeutic INR levels reported. Group A (warfarin transitioned to aspirin) showed the highest bleeding incidence, greater INR variability, and the longest hospitalization duration. Thrombotic events were infrequent and comparably distributed among the groups, with no significant difference in cumulative incidence. A transient prolongation of APTT was observed in Group C (DAPT transitioning to aspirin) without an associated increase in clinical bleeding events.

## Discussion

This study provides a comprehensive evaluation of three distinct antithrombotic strategies following TAVI, highlighting the delicate balance between bleeding and thromboembolic risk. While overall fatal bleeding and thrombotic event did not differ significantly between groups, clinically meaningful differences in bleeding incidence, hospital stay durations, and INR stability were observed, underscoring the importance of individualized antithrombotic management.

Group A (warfarin transitioned to aspirin) demonstrated the highest bleeding incidence (37.5%), with major bleeding events exclusively occurring in patients with supratherapeutic INR levels (>3.5). Longitudinal INR data reveal greater variability in Group A, suggesting that poor INR control significantly contributed to bleeding complications. These findings are consistent with previous studies highlighting the challenges of VKA therapy in elderly patients due to dietary variability, polypharmacy, and metabolic differences. Although transfusion requirements did not differ significantly across groups, Group A had the highest (45.8%), further supporting the clinical impact of bleeding events. Given the irreplaceable role of VKAs in managing mechanical valves recipients and individuals with elevated thrombogenic risk profiles (Van Mieghem et al., 2021), our findings underscore the imperative for meticulous coagulation parameter surveillance and personalized therapeutic following TAVI. Elderly populations receiving VKA therapy face unique challenges stemming from nutritional vitamin K variability, pharmacokinetic interactions inherent to multimorbidity polypharmacy, and genetically determined metabolic heterogeneity (Holbrook et al., 2012). These factors collectively lead to suboptimal anticoagulation control and heightened susceptibility to bleeding complications. Nevertheless, VKAs remains the gold standard for anticoagulation in specific high-risk populations, including patients with mechanical prosthetic valves and those with high thromboembolic burden, where NOACs are contraindicated or insufficiently validated (Vahanian et al., 2021). In these clinical contexts, stringent INR monitoring, patient education on dietary and medication adherence, and individualized dose adjustment are critical to minimize bleeding risk and optimize therapeutic outcomes post-TAVI. Group B (warfarin transitioned to rivaroxaban) demonstrated optimal safety metrics, with no bleeding events, superior anticoagulation stability during the initial treatment phase, and reduced hospitalization duration. These findings align with existing evidence that NOACs, including rivaroxaban, may offer a safer alternative to VKAs by reducing bleeding risk without compromising thromboembolic protection (Van Mieghem et al., 2021; Kuno et al., 2020). However, two ischemic strokes occurred in Group B, highlighting the need for cautious risk assessment, especially in TAVI patients with pre-existing arterial embolization (Didier et al., 2021).

These findings are generally consistent with results from major randomized controlled trials in the TAVI population. For instance, the GALILEO trial demonstrated that a rivaroxaban-based strategy resulted in higher rates of thromboembolic protection compared to antiplatelet therapy alone, but was also associated with significantly increased bleeding and mortality risks, ultimately leading to early trial termination (Dangas et al., 2020). Similarly, the ATLANTIS trial showed that apixaban did not significantly improve thromboembolic outcomes compared to standard care, although bleeding outcomes were more favorable. The ENVISAGE-TAVI AF trial further demonstrated non-inferiority of edoxaban compared to VKAs in thromboembolic efficacy, but with a higher bleeding risk, particularly gastrointestinal hemorrhage (Van Mieghem et al., 2021). In our real-world cohort, transitioning to rivaroxaban was associated with a lower incidence of bleeding without complete elimination of thromboembolic events, echoing the complex trade-off between bleeding and ischemic risks observed in these randomized studies (Collet et al., 2022). Although thrombotic event rates were numerically lower in Group B (14.3%) compared to Group A (20.8%) and comparable to Group C (10.0%), the differences were not statistically significant. Notably, thrombotic events in all groups predominantly occurred during the early postoperative phase, emphasizing that the critical window for both bleeding and thrombosis is concentrated within the first few months after TAVI, irrespective of the antithrombotic regimen. Given the smaller sample size, exclusive use of self-expanding valves, and physician-guided rather than randomized treatment assignment in our study, these findings should be interpreted as complementary to, rather than directly comparable with, prior randomized data.

Group C (dual antiplatelet therapy transitioning to aspirin) experienced both bleeding (30.0%) and thrombotic events (10.0%), including a fatal intracranial hemorrhage and two VTE cases. These findings suggest that while de-escalating to aspirin monotherapy may reduce bleeding risk in low-risk patients, it may inadequately prevent thromboembolic events in higher-risk individuals (Dangas et al., 2020; Brouwer et al., 2020; Nijenhuis et al., 2020; Généreux et al., 2012). Current guidelines increasingly favor SAPT post-TAVI (Maes et al., 2018), particularly for patients without atrial fibrillation or other indications for long-term anticoagulation. However, our findings underscore the necessity of risk stratification before adopting SAPT strategies.

Emerging evidence supports the use of SAPT in selected low-risk patients to minimize bleeding complications without compromising thromboembolic protection. Nevertheless, in patients with elevated thromboembolic risk (such as those with high CHA_2_DS_2_-VA scores, prior VTE, or complex atherosclerotic disease) SAPT alone may be insufficient. In these cases, continued anticoagulation with oral agents should be considered. Therefore, individualized assessment using validated tools such as the CHA_2_DS_2_-VA score (for thrombotic risk), HAS-BLED score (for bleeding risk), and the ARC-HBR criteria (for high bleeding risk) is crucial to guide optimal post-TAVI antithrombotic management. Future strategies may benefit from integrating these structured risk stratification frameworks into clinical decision algorithms to better tailor therapy to patient-specific profiles.

Notably, Group C also demonstrated a significantly prolongation of APTT on postoperative day 3; however, this laboratory finding did not correlate with increased clinical bleeding. The APTT elevation may reflect transient perioperative changes in coagulation dynamics rather than true coagulopathy, although its clinical significance remains uncertain and warrants further investigation.

Importantly, non-inferiority analysis indicated that transitioning to rivaroxaban (Group B) was non-inferior and possibly superior to warfarin (Group A) in bleeding outcomes, while non-inferiority for thrombotic events could not be confirmed. These findings support a tailored approach to antithrombotic management following TAVI, favoring NOAC-based regimens in patients at elevated bleeding risk, provided careful monitoring for early thrombotic events.

Our findings align with current international guidelines. The 2021 ESC/EACTS Guidelines (Vahanian et al., 2021) recommend SAPT as the standard post-TAVI antithrombotic strategy in patients without an indication for long-term anticoagulation, primarily based on bleeding reduction. Similarly, the 2020 ACC/AHA Guidelines (Otto et al., 2020a) advocate SAPT in low thrombotic risk patients. However, both guidelines emphasize the necessity of individualized decision-making based on patient-specific thrombotic and bleeding risk profiles. Our study reinforces these recommendations, highlighting that while SAPT is appropriate for carefully selected low-risk patients, anticoagulation with agents like rivaroxaban may offer advantages in higher-risk cohorts.

Future research should aim to address several critical gaps identified in this and other studies. Longitudinal studies with extended follow-up are needed to determine whether the early benefits of specific anticoagulation strategies persist over time, particularly regarding valve durability, thromboembolic protection, and bleeding outcomes. Additionally, head-to-head comparisons between different NOACs, such as rivaroxaban and apixaban, could provide more precise guidance for agent selection in diverse patient populations.

Personalized approaches to antithrombotic management, leveraging machine learning models that integrate clinical, procedural, and biomarker data, hold promise for optimizing individualized risk stratification and therapeutic decision-making. Furthermore, incorporating biomarkers such as D-dimer levels and platelet function assays into clinical practice may help refine risk-adapted antithrombotic strategies.

Future prospective, randomized trials are needed to validate these findings and further refine antithrombotic strategies in diverse, high-risk TAVI populations. Incorporating structured bleeding risk assessments, such as ARC-HBR criteria, alongside traditional thromboembolic risk stratification tools, may optimize outcomes and personalize therapy in this vulnerable patient group.

## Limitations

This study has several limitations that merit consideration. First, its retrospective design inherently limits the ability to establish causal relationships between antithrombotic regimens and clinical outcomes. Second, the absence of randomization may have introduced selection bias, as treatment assignment was based on physician discretion rather than standardized criteria. Third, the relatively small sample size, particularly in Group B (n = 14), limited the statistical power to detect differences in low-frequency but clinically meaningful critical outcomes such as stroke or fatal bleeding.

Additionally, the study was conducted at two centers within a single geographic region, which may affect generalizability. The follow-up duration of up to 24 months may not fully capture delayed complications including valve thrombosis or recurrent ischemic events. Furthermore, adherence to prescribed antithrombotic therapy was not assessed, which could influence the observed event rates and real-world applicability.

Other potentially confounding variables, including frailty scores, nutritional status, and comprehensive comorbidity indices, were not systematically collected. These omissions may limit risk adjustment and interpretation of group differences. Finally, the lack of routine screening for subclinical events such as silent stroke or leaflet thrombosis may have resulted in underestimation of the true thrombotic burden. Subsequent investigations should prioritize multiethnic population cohorts with prolonged surveillance periods, incorporating advanced imaging biomarkers and pharmacodynamic monitoring to elucidate long-term regimen performance. Integration of machine learning-driven risk prediction models with geriatric assessment tools may enable precision antithrombotic stewardship in this complex patient demographic.

## Conclusion

This study highlights the complexities of antithrombotic management following TAVI, emphasizing the need to balance thrombotic and bleeding risks in a vulnerable population. Transitioning from warfarin to rivaroxaban was associated with a lower bleeding risk and shorter hospitalization compared to traditional VKA-based strategies, although thromboembolic events were not entirely eliminated. In contrast, aspirin monotherapy following dual antiplatelet therapy showed limited thromboembolic protection and a non-negligible bleeding risk, particularly in higher-risk patients. These findings underscore the importance of individualized antithrombotic regimens tailored to patient-specific risk profiles, considering factors such as prior bleeding history, thrombotic risk, and comorbidities. Further randomized studies are warranted to optimize post-TAVI antithrombotic strategies and explore innovative approaches, including risk-adapted regimens or tailored dosing protocols, to improve clinical outcomes while minimizing adverse events.

## Data Availability

The original contributions presented in the study are included in the article/supplementary material, further inquiries can be directed to the corresponding author.
